# Stratification of lncRNA modulation networks in breast cancer

**DOI:** 10.1186/s12920-022-01236-6

**Published:** 2022-05-02

**Authors:** Wen-Hsuan Yu, Chia-Lang Hsu, Chen-Ching Lin, Yen-Jen Oyang, Hsueh-Fen Juan, Hsuan-Cheng Huang

**Affiliations:** 1 Graduate Institute of Biomedical Electronics and Bioinformatics, National Taiwan University, Taipei, Taiwan; 2 Center for Computational and Systems Biology, National Taiwan University, Taipei, Taiwan; 3 Institute of Biomedical Informatics, National Yang Ming Chiao Tung University, No. 155, Sec. 2, Linong Street, Taipei, 112 Taiwan; 4 Department of Medical Research, National Taiwan University Hospital, Taipei, Taiwan; 5 Department of Computer Science and Information Engineering, National Taiwan University, Taipei, Taiwan; 6 Department of Life Science, National Taiwan University, No. 1, Sec. 4, Roosevelt Road, Taipei, 106, Taiwan

**Keywords:** Long non-coding RNA, Gene co-expression network, Association network, Breast cancer

## Abstract

**Background:**

Recently, non-coding RNAs are of growing interest, and more scientists attach importance to research on their functions. Long non-coding RNAs (lncRNAs) are defined as non-protein coding transcripts longer than 200 nucleotides. We already knew that lncRNAs are related to cancers and will be dysregulated in them. But most of their functions are still left to further study. A mechanism of RNA regulation, known as competing endogenous RNAs (ceRNAs), has been proposed to explain the complex relationships among mRNAs and lncRNAs by competing for binding with shared microRNAs (miRNAs).

**Methods:**

We proposed an analysis framework to construct the association networks among lncRNA, mRNA, and miRNAs based on their expression patterns and decipher their network modules.

**Results:**

We collected a large-scale gene expression dataset of 1,061 samples from breast invasive carcinoma (BRCA) patients, each consisted of the expression profiles of 4,359 lncRNAs, 16,517 mRNAs, and 534 miRNAs, and applied the proposed analysis approach to interrogate them. We have uncovered the underlying ceRNA modules and the key modulatory lncRNAs for different subtypes of breast cancer.

**Conclusions:**

We proposed a modulatory analysis to infer the ceRNA effects among mRNAs and lncRNAs and performed functional analysis to reveal the plausible mechanisms of lncRNA modulation in the four breast cancer subtypes. Our results might provide new directions for breast cancer therapeutics and the proposed method could be readily applied to other diseases.

**Supplementary Information:**

The online version contains supplementary material available at 10.1186/s12920-022-01236-6.

## Background

Non-coding RNAs (ncRNAs) are functional RNA molecules transcribed from DNA but not translated into proteins. Non-coding RNAs have been known to make up a majority of transcribed RNAs, in which many different types of regulatory RNAs have been, and continue to be, discovered. Long non-coding RNAs (lncRNAs) are a novel class of RNA molecules defined as transcripts longer than 200 nucleotides that, in many ways, are similar to protein-coding transcripts, except for lack of significant protein-coding potential. On the whole, most parts of the human genome are composed of non-coding genes. Recent studies found that non-coding genes play a significant role in regulating gene expression. Distinct molecular mechanisms allow lncRNAs to activate or repress gene expression, thereby involved in the regulation of cellular and tissue functions. Cancer is a disease with aberrant gene expression, as some studies showed that several cancer risk loci are transcribed into lncRNAs and these transcripts play critical roles in tumorigenesis [1]. In addition, many lncRNAs are deregulated in cancer, and some of them can be important drivers of malignant transformation. Hence some lncRNAs can be tumor markers or potential drug-targets for cancer treatment. Long non-coding RNAs have gained widespread attention nowadays as a new biological regulation, but sorting out what they do is challenging [2].

MicroRNAs (miRNAs) are widely known as a class of non-coding RNA (ncRNA), single-stranded RNAs of 18–25 nucleotides in length [3]. MicroRNAs bind to sequences with partial complementarity on target RNA transcripts, called microRNA response elements (MREs), usually resulting in the repression of target gene expression. MREs are sequences in the 3' untranslated regions (UTRs) of target RNA transcripts and typically have a conserved stretch of 7 nucleotides that are able to base pair with the 5 regions of corresponding miRNAs. In 2011, Pandolfi proposed the competitive endogenous hypothesis [4], according to which, every RNA transcripts can regulate each other by sharing common MREs, which would work as miRNA sponge, thus facilitating translation of the target RNA transcripts, despite the presence of corresponding miRNA. Competing endogenous RNAs (ceRNAs) are new classification of RNAs, which competes with specific mRNA for providing biding sites to the corresponding miRNA. Recently, numerous studies have reported that there exists ceRNA mechanism between protein-coding messenger RNAs and non-coding RNA such as transcribed pseudogenes, lncRNAs and circular RNAs (circRNAs). They co-regulate each other by competing for binding to shared miRNAs [5]. Competing endogenous RNA interactions form a multilayered network that regulates gene expression in various biological pathways. Recent reports have demonstrated that ceRNA networks regulate essentially all known biological processes, while their functions remain to be explored yet.

In general, miRNAs are negative regulators of gene expression, decreasing the stability of target RNAs. Additionally, miRNA also regulates lncRNA. However, on the basis of ceRNA hypothesis, all types of RNA transcripts can actively communicate to each other to regulate their respective expression levels by using MREs [4]. In principle, overexpression of ceRNAs increases the concentration of specific MREs, therefore, leading to the increased expression of mRNA. Our knowledge of lncRNA can act as miRNA sponges, which then reduce the amount of miRNA available to target mRNAs. In addition, lncRNA may possess ceRNA activity in various cancers, and have been implicated in human development. Thus, we can use this regulation to investigate the functions of lncRNA. Recent studies have linked specific lncRNAs to development, cancer, pain, inflammation, and other important biological processes and phenomena. Furthermore, lncRNAs were found to be widely expressed and often dysregulated in cancers, while their functions and mechanism remain to be explored yet. Recently, a new class of RNAs was discovered, which co-regulate each other by competing for binding to shared microRNAs and described as competing endogenous RNAs (ceRNAs). CeRNAs are widely implicated in many biological processes and have been found to be important regulator in many types of cancer [6]. Besides, ceRNA has been proposed to explain the complex relationships among lncRNAs and mRNAs to compete for miRNA binding. In this study, we collected a large-scale gene expression dataset for cancer, and proposed an analysis framework to integrate them. We expect to uncover the underlying ceRNA modules and the key modulatory lncRNAs in different cancer types.

## Results

### Construction of bipartite co-expression network

**Fig. 1:**
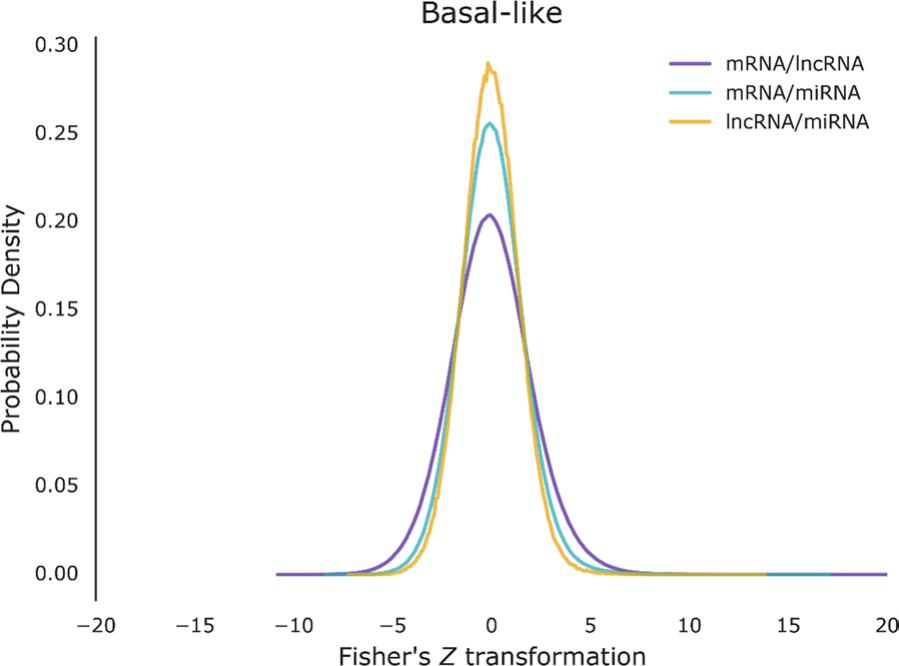
Distribution of Fisher’s Z transformation. Distribution of Fisher transformation for Spearman correlation coefficients between all mRNA/lncRNA pairs, mRNA/miRNA pairs and lncRNA/miRNA pairs in BRCA Basal-like

We analyzed a large dataset of BRCA profiles for mRNA, lncRNA and miRNA expression. These expression data were obtained from Genomic Data Commons (GDC) data portal. The BRCA patient cohort can be subdivided into four breast cancer subtypes as reported previously [7, 8], so we divided the samples into four subtypes for our further analysis (Table 1). After data preprocessing, we computed the Fisher’s z transformation of Spearman rank correlation coefficients among lncRNAs, mRNAs and miRNAs for each cancer subtype, respectively. The z-score distribution curves are all unimodal, symmetric and centered at zero (Fig. 1, Additional file 1: Fig. S1-S3). We used the scale-free topology criterion to choose a cut-off z-score value for construction of gene co-expression networks [9]. Here, we selected interactions with the absolute value of z-score greater than or equal to the cut-off z-score value. The RNA-RNA pairs with sparse interactions were removed, and all the remaining interactions were used to construct the bipartite networks of lncRNA-mRNA, mRNA-miRNA, and miRNA-lncRNA (Additional file 1: Fig. S4a). As a result, we got three bipartite co-expression networks in four subtypes, respectively.

**Table 1:** Summary of analyzed BRCA cohort and data sets

Cancer subtype	Samples	mRNAs	lncRNAs	miRNAs
Basal-like	167	12,336	808	491
Her2 type	115	12,323	785	491
Luminal A	405	12,518	884	491
Luminal B	287	12,123	880	491

### The interaction-profile similarity between genes and identification of coherent association

**Fig. 2:**
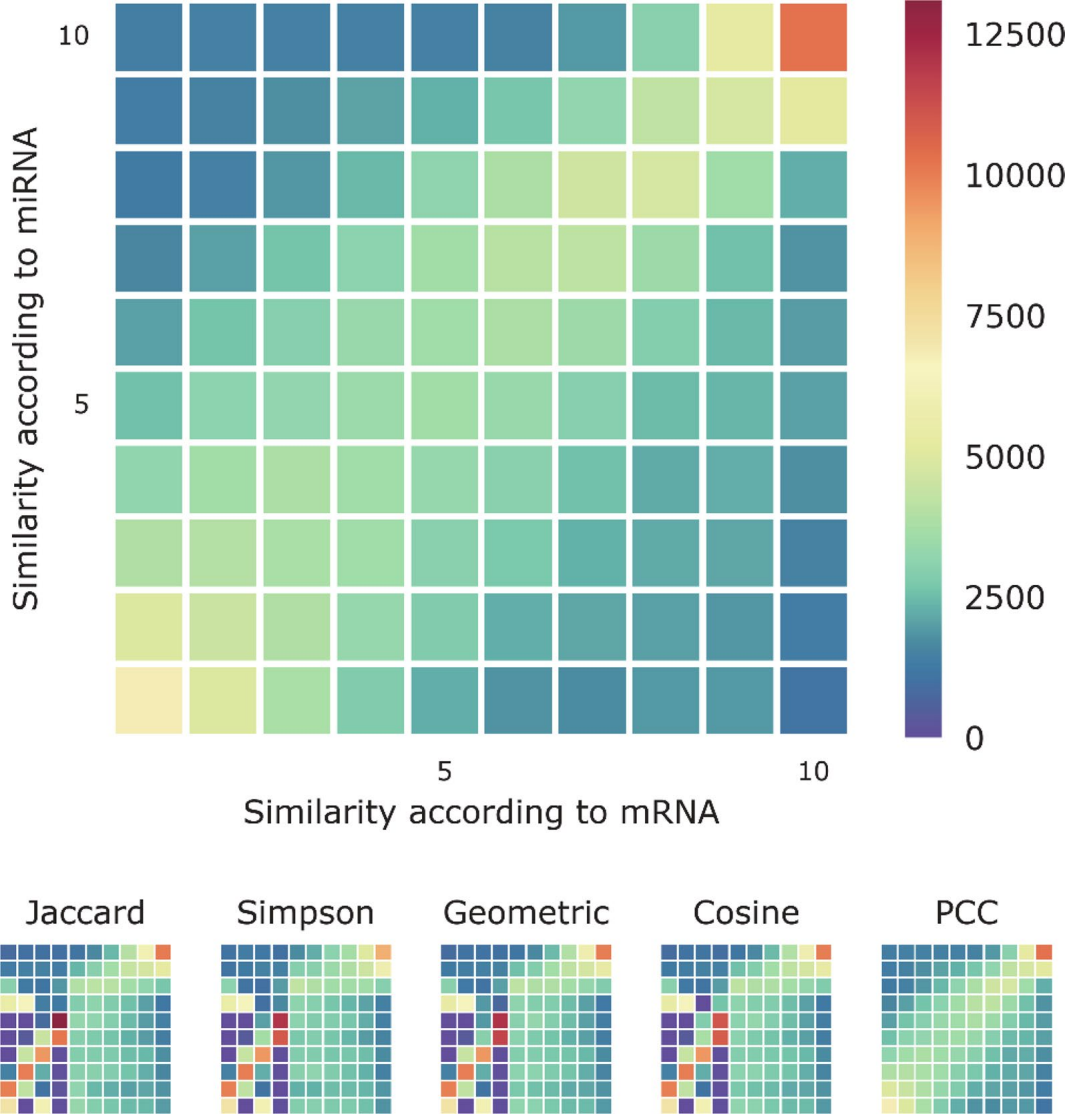
Visualized density histogram of lncRNA (Basal-like). Density histogram indicated five types of association indices to measure shared mRNA and miRNA nodes between two lncRNA nodes in bipartite networks. The top histogram is for association indices computed using the Pearson correlation coefficient (PCC). The bottom five histograms are for association indices computed using the Jaccard index, the Simpson index, the geometric index, the cosine index, and PCC, respectively (from left to right)

Metrics known as association indices can be used to quantify interaction-profile similarity. In 2013, Walhout and co-workers described an overview of commonly used association indices [10], and compared the performance of different methods in various types of analyses for biological networks. In addition, network analysis can identify modules, composed of genes with similar interaction profiles, that can imply functional relationships between genes. The interaction-profile similarities between genes can be calculated using the association indices. However, different association indices provide different values of the interaction-profile similarity. Here, we focused on five commonly used measures to quantify interaction-profile similarity (See Methods). For a pair of lncRNAs, we can measure their association by calculating their node similarity (association index) in the lncRNA-miRNA bipartite co-expression network; we can also measure the association by their similarity in the lncRNA-mRNA bipartite network. To compare the lncRNA-lncRNA associations (similarities) calculated from the two different bipartite networks, we displayed the distributions of all the lncRNA pairs as density histograms (Fig. 2; Additional file 1: Fig. S4b) with their coordinates set to their similarities calculated by lncRNA-mRNA bipartite network and lncRNA-miRNA network, respectively. Our results showed that the lncRNA-lncRNA pairs with high similarity in one bipartite network (lncRNA-mRNA) tended to have high similarity in another bipartite network (lncRNA-miRNA), and the pairs with low similarity in one bipartite network often had low similarity in another bipartite network contrariwise.

**Fig. 3:**
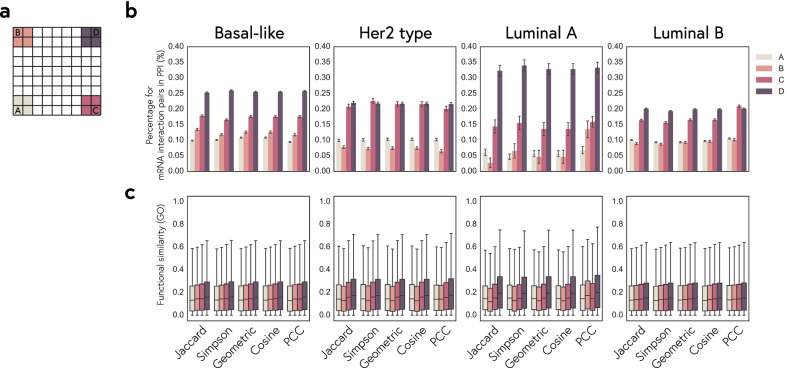
Identification of coherent association. **a** Definition of four areas A–D in the interaction profile similarity map for association indices between two mRNAs measured by shared lncRNAs versus the indices measured by shared mRNAs. This map of mRNAs is defined similar to the density histograms of lncRNAs in Fig. 2. **b** Percentages of gene pairs with protein–protein interactions (PPI) among the associated mRNAs selected in the area A, B, C, or D. Each plot shows the analysis results of each breast cancer subtype obtained using five different association indices, respectively. **c** GO functional similarity between associated mRNA pairs selected in each area using five different association indices for the four breast cancer subtypes

Network modules are groups of nodes with high interaction-profile similarity and the nodes in the same module tend to have related biological functions. Because of this, we can identify network modules using association indices. Since the functions and molecular mechanisms of most lncRNAs still remain unknown, we used mRNA as a proxy to determine the criteria for construction of coherent association network and evaluate the functional association of mRNA pairs with similar interaction profiles. The mRNA-mRNA similarity was assessed by their association indexes in the mRNA-lncRNA bipartite co-expression network and also by mRNA-miRNA bipartite network. Presumably the mRNA pairs having high similarity in both association networks share similar biological functions and are likely involved in the same biological pathways. We defined four areas in the coordinate system for interaction-profile similarity (Fig. 3a) and evaluated their performance by protein–protein interaction analysis and GO functional similarity. Later we would apply the same selection criteria to construct association network of lncRNAs.

In protein–protein interaction analysis, we calculated the percentages for each set of mRNAs with PPIs. It indicates that these mRNAs have related biological functions if they have high percentage of interaction in PPIs. As a result of analysis, we observed the percentage of area D is higher than other areas in four subtypes (Fig. 3b). However, we can identify functionally related mRNAs by calculating GO functional similarity. We compared the functional similarity to establish functional relationship between each pair of mRNAs that belong to four areas (Fig. 3c). As a result, we found pairs of mRNAs that belong to the area D corresponds to highly similar functions than other areas in four subtypes. Overall, area D performs the best, and area A performs the worst. Thus, we focused on gene pairs having related biological functions, which we called ‘coherent association’. The pairwise association indices values in area D are for coherent association. We applied this selection criteria to construct the association network of lncRNAs.

### Association networks of lncRNAs

**Fig. 4:**
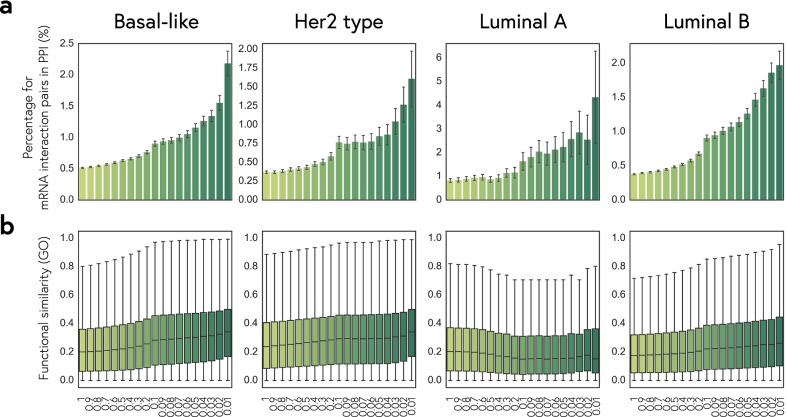
Evaluating functional performance for identifying thresholds in BRCA subtypes. We integrated five association indices and compared the characteristics of coherently associated gene pairs selected with different thresholds. **a** Percentages of gene pairs with PPI for different selection thresholds of association indices. **b** GO functional similarity of the coherently associated gene pairs selected by different thresholds of association indices

**Fig. 5:**
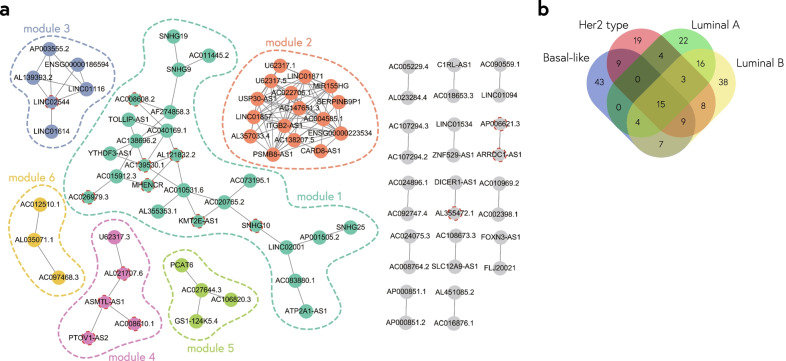
Coherent association network. **a** Coherent association network in BRCA Basal-like. Network for 87 lncRNAs by high interaction-profile similarity, where nodes represent lncRNAs. Nodes are colored by modules and gray nodes represent lncRNAs unassigned to a module. The red border indicates lncRNAs found in four subtypes. **b** Venn diagram showing the number of shared and unique lncRNAs from coherent association network under different subtypes

In the above analysis, we used five methods to calculate interaction-profile similarity between genes and evaluate their performance on inferring functional association. Thereby, we combined five association indices and chose to focus on high-confidence interactions that confirmed by three association indices. We performed PPIs analysis and the GO functional similarity to compare the performance of associated mRNA pairs selected from different thresholds (Fig. 4). We chose to focus on high-confidence interactions that were confirmed by three association indices and pairwise association indices in the top 0.05 percent. The results of coherent association network in four subtypes showed modular structures (Fig. 5a; Additional file 1: Fig. S5-S7). Interestingly, we found that only 15 lncRNAs appeared in all the coherent association networks (Fig. 5b).

### Comparison of functional modules between subtypes

**Fig. 6:**
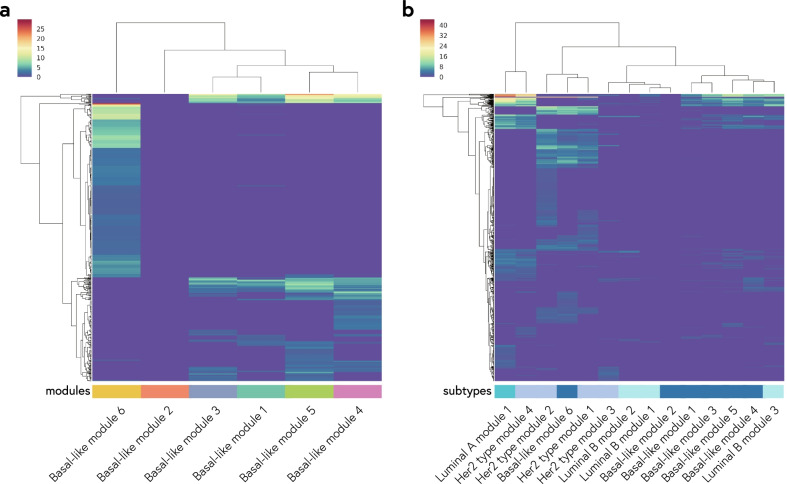
Functional association among modules. **a** Hierarchically-clustered heatmap of gene ontology categories enrichment analysis across Basal-like modules. **b** Hierarchically-clustered heatmap of gene ontology categories enrichment analysis across fourteen modules. Only categories with an adjusted FDR-q-value of less than 0.05 in at least one module are shown

To understand the biological role of lncRNAs, we performed Gene Ontology (GO) enrichment analysis using mRNAs co-expressed with the lncRNAs in each module. We focus on the top six modules of lncRNA coherent association network in the BRCA Basal-like. As a result, the module 1, 3, 4 and 5 are enriched in macromolecule metabolic process, cellular macromolecule metabolic process, whereas the module 6 is enriched in regulation of immune system process, positive regulation of immune system process and immune system process. The result showed no significant (FDR-q-value < 0.05) biological process GO term was identified for the module 2 (Fig. 6a and Table 2). We further performed gene ontology categories enrichment analysis across subtypes. In BRCA Her2 type, the module 1 and 2 are enriched in regulation of immune system process and immune system process. The module 3 is enriched in regulation of cell migration and regulation of cell motility, whereas the module 4 is enriched in macromolecule metabolic process and cellular macromolecule metabolic process. The biological process GO term enriched for BRCA Luminal-A included macromolecule metabolic process, cellular macromolecule metabolic process, and organelle organization. In BRCA Her2 type, the module 1 and 3 are enriched in macromolecule metabolic process and cellular component organization, whereas the module 2 is enriched in mitotic nuclear division, cell cycle and nuclear division. We further compared biological process GO term across fourteen modules that belong to the four subtypes (Fig. 6b). Dendrogram showing results of an agglomerative hierarchical cluster analysis. We see that at a high height, there seems to be two distinct groups which first emerge. And the right-hand group has three groups in itself. Therefore, the fourteen modules can be divided into four clusters. We noticed that all four clusters contained at least two subtypes.

**Table 2:** Top 10 significantly enriched Gene Ontology terms

Term	Name	*P* value	FDR *Q* value
*Module 1*			
GO:0043170	Macromolecule metabolic process	1.17E−13	6.83E−10
GO:0044260	Cellular macromolecule metabolic process	5.79E−13	1.69E−09
GO:0033108	Mitochondrial respiratory chain complex assembly	1.23E−11	2.39E−08
GO:0034622	Cellular protein-containing complex assembly	8.75E−11	1.27E−07
GO:0010257	NADH dehydrogenase complex assembly	1.44E−10	1.68E−07
GO:0032981	Mitochondrial respiratory chain complex I assembly	1.44E−10	1.68E−07
GO:0006807	Nitrogen compound metabolic process	4.17E−10	3.47E−07
GO:0044237	Cellular metabolic process	5.69E−10	4.14E−07
GO:0090304	Nucleic acid metabolic process	7.70E−10	4.98E−07
GO:0044238	Primary metabolic process	1.11E−09	6.48E−07
*Module 3*			
GO:0043170	Macromolecule metabolic process	2.02E−18	1.07E−14
GO:0044260	Cellular macromolecule metabolic process	2.74E−18	7.26E−15
GO:0006807	Nitrogen compound metabolic process	1.57E−15	2.76E−12
GO:0044237	Cellular metabolic process	2.18E−15	2.89E−12
GO:0044238	Primary metabolic process	3.12E−14	3.30E−11
GO:0044267	Cellular protein metabolic process	4.53E−14	4.00E−11
GO:0071704	Organic substance metabolic process	5.56E−13	4.21E−10
GO:0008152	Metabolic process	1.16E−12	7.69E−10
GO:0006996	Organelle organization	2.28E−12	1.34E−09
GO:0043412	Macromolecule modification	3.38E−11	1.79E−08
*Module 4*			
GO:0043170	Macromolecule metabolic process	4.77E−20	2.38E−16
GO:0044260	Cellular macromolecule metabolic process	6.52E−19	1.62E−15
GO:0044237	Cellular metabolic process	9.22E−17	1.53E−13
GO:0006996	Organelle organization	1.84E−16	2.29E−13
GO:0006807	Nitrogen compound metabolic process	3.85E−16	3.84E−13
GO:0071704	Organic substance metabolic process	3.87E−15	3.22E−12
GO:0044238	Primary metabolic process	5.08E−15	3.62E−12
GO:0044267	Cellular protein metabolic process	1.02E−14	6.34E−12
GO:0008152	Metabolic process	1.03E−14	5.70E−12
GO:0034641	Cellular nitrogen compound metabolic process	1.66E−11	8.26E−09
*Module 5*			
GO:0043170	Macromolecule metabolic process	2.69E−28	2.01E−24
GO:0044260	Cellular macromolecule metabolic process	2.73E−25	1.02E−21
GO:0006807	Nitrogen compound metabolic process	2.51E−22	6.25E−19
GO:0044237	Cellular metabolic process	1.21E−20	2.27E−17
GO:0044267	Cellular protein metabolic process	5.58E−19	8.33E−16
GO:0071840	Cellular component organization or biogenesis	1.29E−17	1.61E−14
GO:0044238	Primary metabolic process	1.54E−17	1.64E−14
GO:0008152	Metabolic process	4.73E−17	4.41E−14
GO:0006996	Organelle organization	1.39E−16	1.15E−13
GO:0071704	Organic substance metabolic process	2.39E−16	1.78E−13
Module 6			
GO:0002682	Regulation of immune system process	1.70E−34	1.05E−30
GO:0002684	Positive regulation of immune system process	1.48E−28	4.56E−25
GO:0002376	Immune system process	7.55E−27	1.55E−23
GO:0034097	Response to cytokine	4.11E−16	6.34E−13
GO:0050776	Regulation of immune response	4.29E−16	5.29E−13
GO:0002694	Regulation of leukocyte activation	5.63E−16	5.79E−13
GO:0051249	Regulation of lymphocyte activation	2.09E−15	1.84E−12
GO:0050865	Regulation of cell activation	1.21E−14	9.34E−12
GO:0051707	Response to other organism	3.28E−14	2.25E−11
GO:0043207	Response to external biotic stimulus	3.28E−14	2.25E−11

## Discussion

We proposed a novel network analysis method which is suitable to explore the lncRNA modulation. Our strategy incorporates gene expression profiles, patient cohort clustering, computational interaction-profile similarities and gene function prediction to identify the lncRNA network modules and reveal the plausible modulatory mechanisms of lncRNA, mRNA and miRNA co-regulatory networks in human breast invasive carcinoma. Long non-coding RNAs are of growing interests and more scientists attach importance to research on their functions. But most of their functions are still left to further study. The competing endogenous RNAs among mRNAs and lncRNAs by competing for binding with shared miRNAs may help find out the involved biological functions of lncRNAs. Furthermore, we consider that many cancers have multiple subtypes with different causes and clinical outcomes. As large amount of cancer genomics and transcriptomics data are emerging, applying our proposed method to available data will allow us to find key lncRNA modules and reveal their functions in different cancer subtype.

## Conclusions

In conclusion, we have developed a novel method to identify lncRNA network modules in each subtype of breast invasive carcinoma and found candidate key modulatory lncRNAs in the gene regulatory network. Our approach can be readily applied to other cancers or diseases. We might further provide new insights into their underlying mechanisms and suggest new therapeutic targets or approaches. That will benefit the future biomedical research and make contribution to the understanding of cancer diseases.

## Methods

### Data preprocessing

The HTSeq-FPKM expression data and the isoform expression data of human breast invasive carcinoma (BRCA) were obtained from Genomic Data Commons (GDC) data portal (Data Release 10, downloaded on December 25, 2017; https://portal.gdc.cancer.gov/). We obtained gene annotation and mature miRNA information from Ensembl BioMart (Ensemble Genes 90, GRCh38.p10, downloaded on November 7, 2017; https://asia.ensembl.org/index.html) and the miRBase Sequence Database (Release 21, downloaded on June 27, 2017; http://www.mirbase.org/), respectively. For the two expression data sets, we kept only biological features with at least 80% of non-zero values over all samples and performed the upper quartile normalization. To allow log transformation, we identified the minimum non-zero element in each expression data set before adding it element-wise to the original expression data set. The RNA-Seq expression data were then ${log}_{2}$ transformed. We further removed samples that are outliers (beyond two standard deviations away from the mean value) from the data set. For HTSeq-FPKM expression data set, we separated protein coding genes and lncRNAs (including lincRNAs and antisense RNAs) based on human gene annotation from Ensembl. In total, we analyzed 1,061 patients with 4,359 lncRNAs, 1,061 patients with 16,517 mRNAs and 1,034 patients with 534 miRNAs for BRCA data sets.

### Identification of BRCA subtypes

For BRCA cohort subtypes, we identified each sample based on Ashouri et al. [8]. The BRCA cohort they identified were subdivided into the four established PAM507 subtypes. Here, our BRCA cohort included 167 samples classified as Basal-like, 115 samples classified as Her2 type, 405 samples classified as Luminal A, and 287 samples classified as Luminal B. To filter mRNAs and lncRNAs with low expression across most samples in each subtype, we removed mRNAs and lncRNAs that were median FPKM values <  = 0.7 for downstream analyses (Table 1).

### Gene co-expression analysis

We selected the highly-correlated lncRNA/mRNA pairs, lncRNA/miRNA pairs and mRNA/miRNA pairs in four subtypes. We computed z-score which is the Fisher transformation of Spearman's rank correlation coefficients among lncRNAs, mRNAs and miRNAs for the cancer subtypes, respectively. The Spearman correlation coefficient is a statistical measure of the strength of a monotonic relationship between paired data. In a sample it is denoted by *rs* and is by design constrained as

1 $$-1\le {r}_{s}\le 1$$

And it is defined as the Pearson correlation coefficient between the ranked variables. Furthermore, unlike Pearson correlation coefficient, there is no requirement of normality and hence it is a nonparametric statistic. Now

2 $${r}_{s}=1-\frac{6\sum {{d}_{i}}^{2}}{n\left({n}^{2}-1\right)}$$

where $n$ is a sample of size, the $n$ raw scores ${X}_{i}, {Y}_{i}$ are converted to ranks ${x}_{i}, {y}_{i}$, and ${d}_{i}={x}_{i}-{y}_{i}$ is the difference between ranks.

Then we computed z-score using the Fisher transformation of (${r}_{s}$):

3 $$F\left({r}_{s}\right)=\frac{1}{2}\mathrm{ln}\frac{1+{r}_{s}}{1-{r}_{s}}$$

where ${r}_{s}$ is the sample Spearman rank correlation coefficient. And then

4 $$z=\sqrt{\frac{n-3}{1.06}}F\left({r}_{s}\right)$$

where *z* is a z-score for ${r}_{s}$ which approximately follows a standard normal distribution, and *n* is the sample size.

### Construction of bipartite co-expression networks

A bipartite network describes the interactions between two different types of nodes (X-type and Y-type), with edges connecting only nodes of different types. We selected the highly-correlated lncRNA/mRNA, lncRNA/miRNA and mRNA/miRNA pairs to construct three bipartite co-expression networks (Additional file 1: Figure S4a) in four subtypes. To choose a cut-off z-score value we used the Scale-free Topology Criterion [9], which applies the linear regression model fitting index (${R}^{2}$) to quantify how well a network satisfies a scale-free topology. If parameter values lead to an ${R}^{2}$ value close to 1 may lead to networks with very few connections. Here we only consider those z-score values that lead to a bipartite network satisfying scale-free topology at least approximately: ${R}^{2}$ > 0.8 and the slope of the regression line between ${\mathrm{log}}_{10}\left(p\left(k\right)\right)$ and ${\mathrm{log}}_{10}(k)$ should be greater than -2 but less than -0.5.

### Association indices and networks

We calculated the interaction-profile similarities between genes. Using networks to measure similarity between genes and especially focus on bipartite networks that connect X-type nodes to Y-type nodes. In bipartite networks, association indices can be used to measure shared Y-type nodes between two X-type nodes, or vice versa. Here, we calculated the association indices between mRNA-mRNA pairs in mRNA-lncRNA and mRNA-miRNA bipartite network, and the same for lncRNA-lncRNA pairs and miRNA-miRNA pairs.

We focused on the following five measures to quantify interaction-profile similarity [10], including the Jaccard index, the Simpson index, the geometric index, the cosine index, and the Pearson correlation coefficient. For example, we used five association indices to measure interaction-profile similarity between X-type nodes A and B.

The Jaccard index is the proportion of shared nodes between A and B relative to the total number of nodes connected to A or B:

5 $$\frac{\left|N\left(A\right)\cap N\left(B\right)\right|}{\left|N\left(A\right)\cup N\left(B\right)\right|}$$

where $N\left(A\right)$ is defined as the number of nodes with which A interacts (similarly for $N\left(B\right)$).

The Simpson index is the proportion of shared nodes relative to the degree of the least-connected node:

6 $$\frac{\left|N\left(A\right)\cap N\left(B\right)\right|}{\mathrm{min}\left(\left|N\left(A\right)\right|, \left|N\left(B\right)\right|\right)}$$

The geometric index corresponds to the product of the proportion of shared nodes between A and B:

7 $$\frac{{\left|N\left(A\right)\cap N\left(B\right)\right|}^{2}}{\left|N\left(A\right)\right|\cdot \left|N\left(B\right)\right|}$$

The cosine index is the geometric mean of the proportions of shared nodes between A and B:

8 $$\frac{\left|N\left(A\right)\cap N\left(B\right)\right|}{\sqrt{\left|N\left(A\right)\right|\cdot \left|N\left(B\right)\right|}}$$

The Pearson correlation coefficient is the correlation between the interaction profiles of A and B:

9 $$\frac{\left|N\left(A\right)\cap N\left(B\right)\right|\cdot {n}_{y}-\left|N\left(A\right)\right|\cdot \left|N\left(B\right)\right|}{\sqrt{\left|N\left(A\right)\right|\cdot \left|N\left(B\right)\right|\cdot \left({n}_{y}-\left|N\left(A\right)\right|\right)\cdot \left({n}_{y}-\left|N\left(B\right)\right|\right)}}$$

where ${n}_{y}$ is the total number of Y-type nodes in the network.

We expected to uncover the underlying complex regulatory relationships among lncRNAs, mRNAs and miRNAs. The association network is a network in which two nodes of the same type are connected by an edge with their similarity. It enables the comparison of pairs of nodes across networks using the integration of different types of networks, whether pairs of nodes with similar interaction profiles in one bipartite network are also similar in another bipartite network. An illustration of the analysis workflow is shown in Additional file 1: Figure S4b.

### Protein–protein interaction analysis

We listed pairwise genes in four areas and calculated the percentage of pair in PPIs database, respectively. The PPIs information were obtained from ConsensusPathDB-human (downloaded on July 31, 2017; http://cpdb.molgen.mpg.de/). In total, ConsensusPathDB-human contains 272,998 protein interactions. All binary PPIs have an aggregated confidence score that was computed as a consensus score across the six methods for judging interaction confidence [11]. We only used 92,728 high-quality PPI interactions whose consensus score > 0.95.

### Functional similarity analysis

The Gene Ontology (GO) is a standard vocabulary of functional terms and gene product attributes across all species. The GO is divided into three orthogonal ontologies, biological process, molecular function, and cellular component. Gene products are functionally similar if they have similar molecular functions and biological processes. GO annotations can be used as a measure of functional similarity between gene products. We used $funSim$ to assess the functional relationship between two genes [12]. Lengauer and co-workers provide a new measure of similarity between GO terms. This new measure is based on Lin’s [13] and Resnik’s [14, 15] definitions, called ${sim}_{Rel}$. We also measured and compared functional similarity between genes in four areas, respectively.

## Supplementary Information

**Additional file 1: Figure S1**. Distributions of Fisher’s Z transformation for BRCA Her2 type. **Figure S2**. Distributions of Fisher’s Z transformation for BRCA Luminal A. **Figure S3**. Distributions of Fisher’s Z transformation for BRCA Luminal B. **Figure S4**. Illustrations of three kinds of bi-partite co-expression networks and the workflow of the proposed analysis method. **Figure S5**. Coherent association network in BRCA Her2 type. **Figure S6**. Coherent association network in BRCA Luminal A. **Figure S7**. Coherent association network in BRCA Luminal B.

## Data Availability

The RNA-seq dataset is available via the Genomic Data Commons data portal (https://portal.gdc.cancer.gov/).

## Abbreviations

lncRNAs: Long non-coding RNAs
ceRNAs: Competing endogenous RNAs
mRNAs: Messenger RNAs
miRNAs: MicroRNAs
BRCA: Breast invasive carcinoma
ncRNAs: Non-coding RNAs
MREs: MicroRNA response elements
UTRs: Untranslated regions
circRNAs: Circular RNAs
GDC: Genomic data commons
PAM: Prediction Analysis of Microarray
SCC: Spearman's rank correlation coefficients
GO: Gene ontology
PPIs: Protein–protein interactions
