# Supplementary figures and images for: Stratification of lncRNA modulation networks in breast cancer
Source: BMC Med Genomics. 2022 May 2;14(Suppl 3):300. doi: 10.1186/s12920-022-01236-6 (PMC9059351; doi:10.1186/s12920-022-01236-6)

Additional file 1

Figure S1.

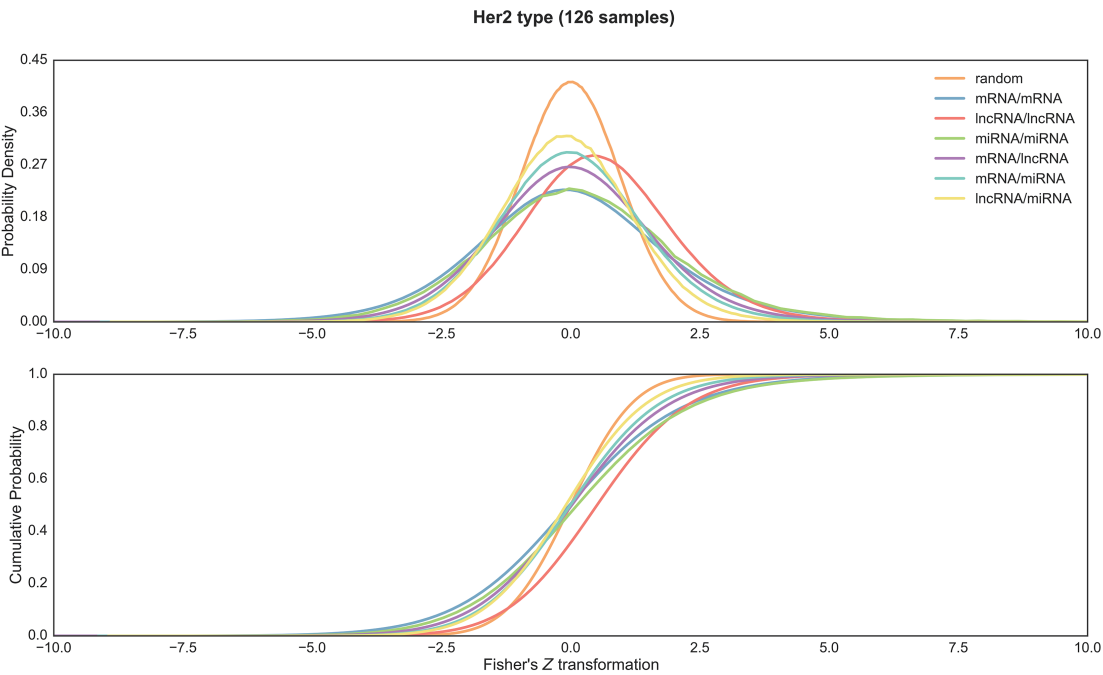

Figure S2.

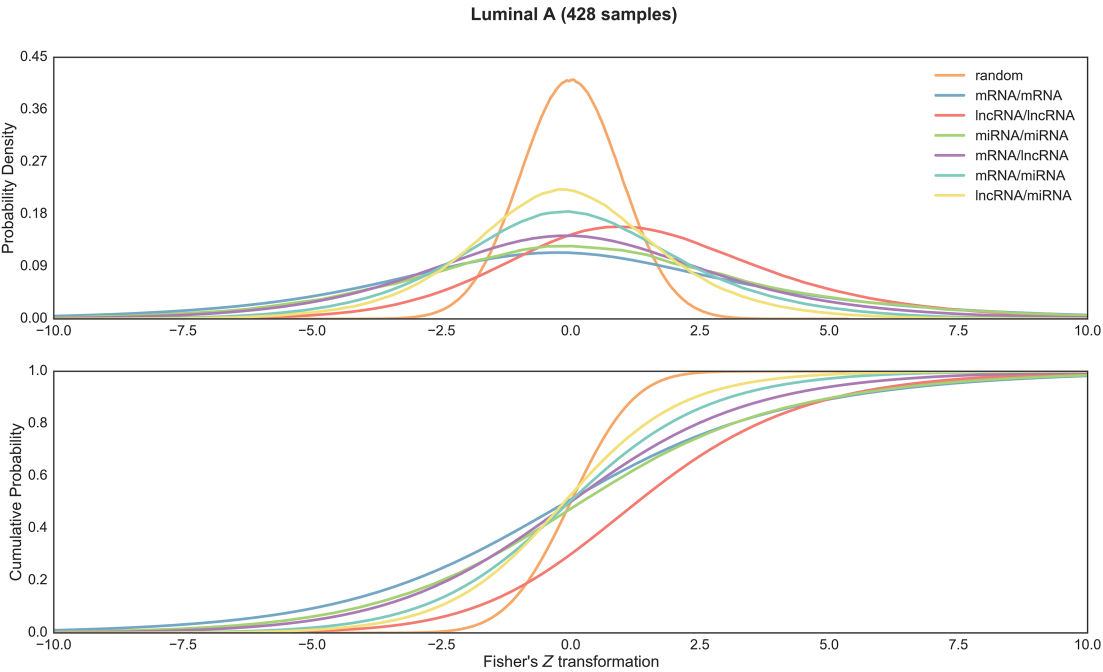

Figure S3.

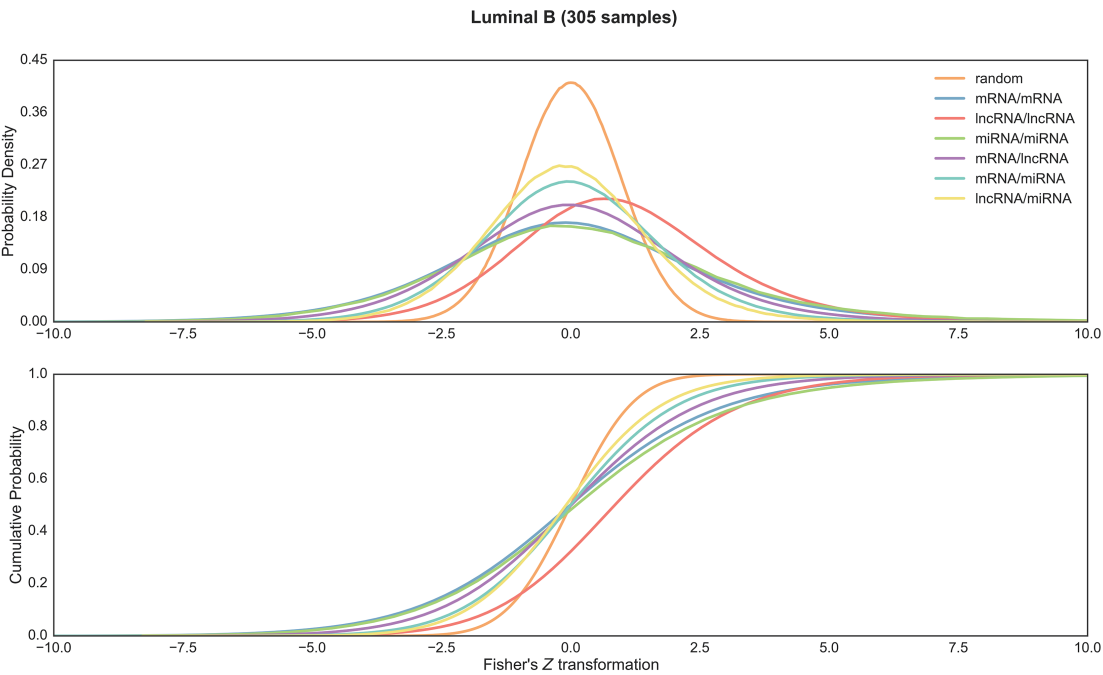

Figure S4.

a

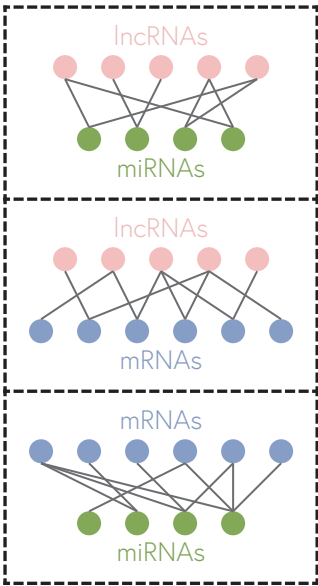

b

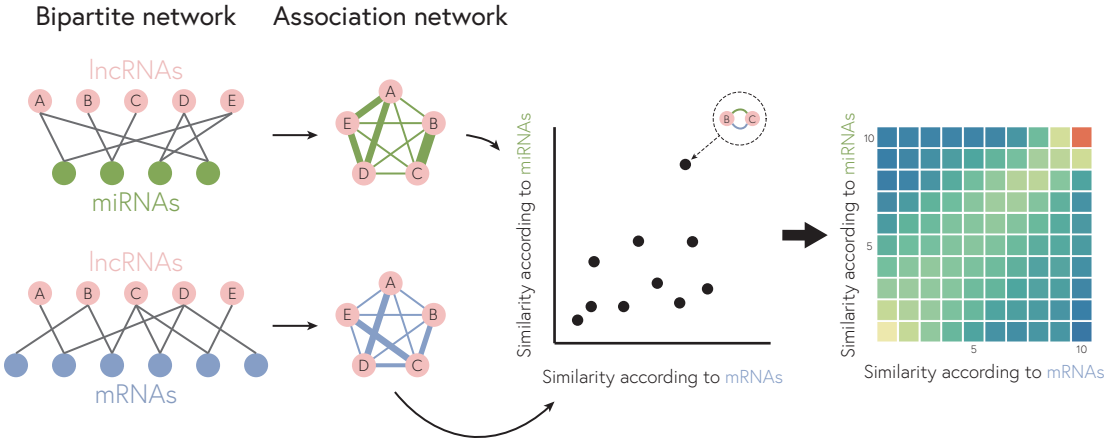

Figure S5.

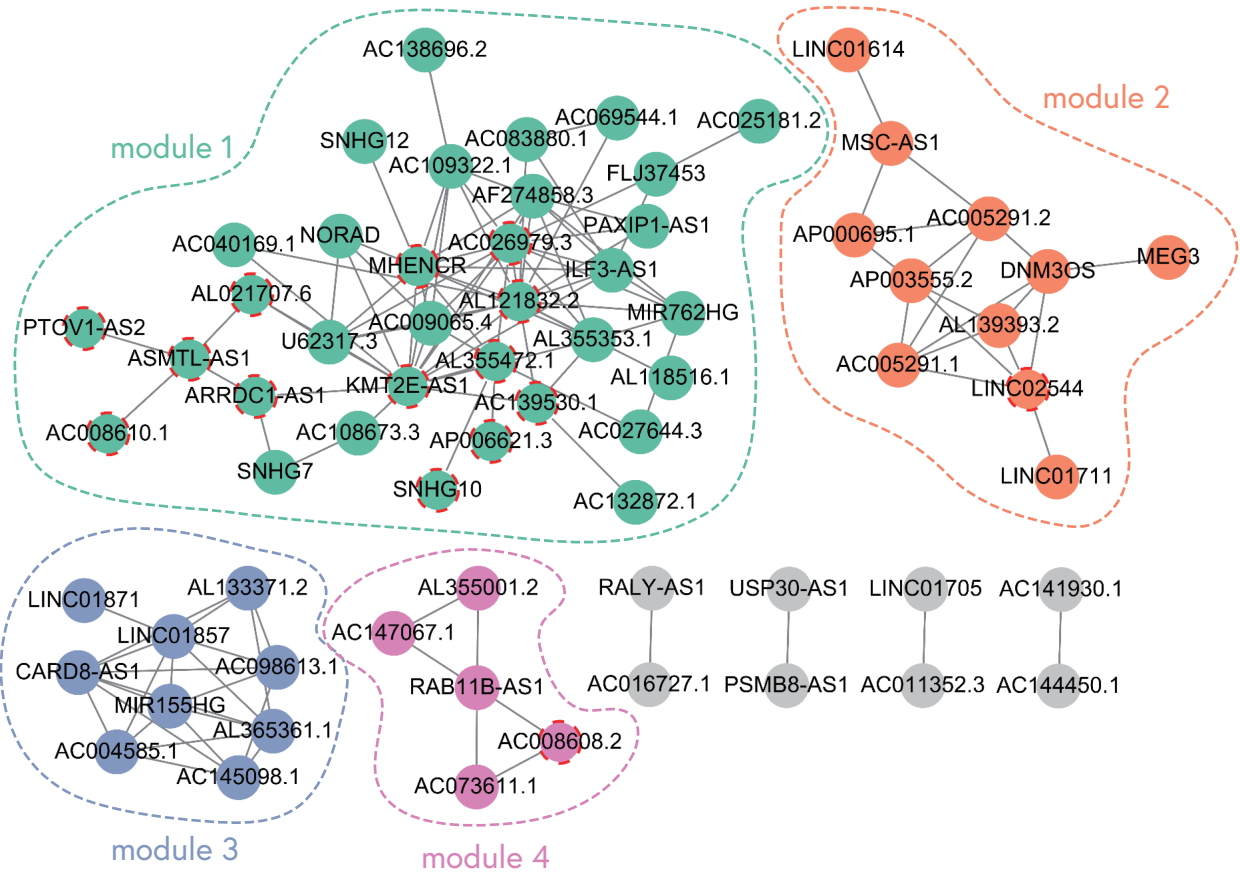

**Figure S6.**

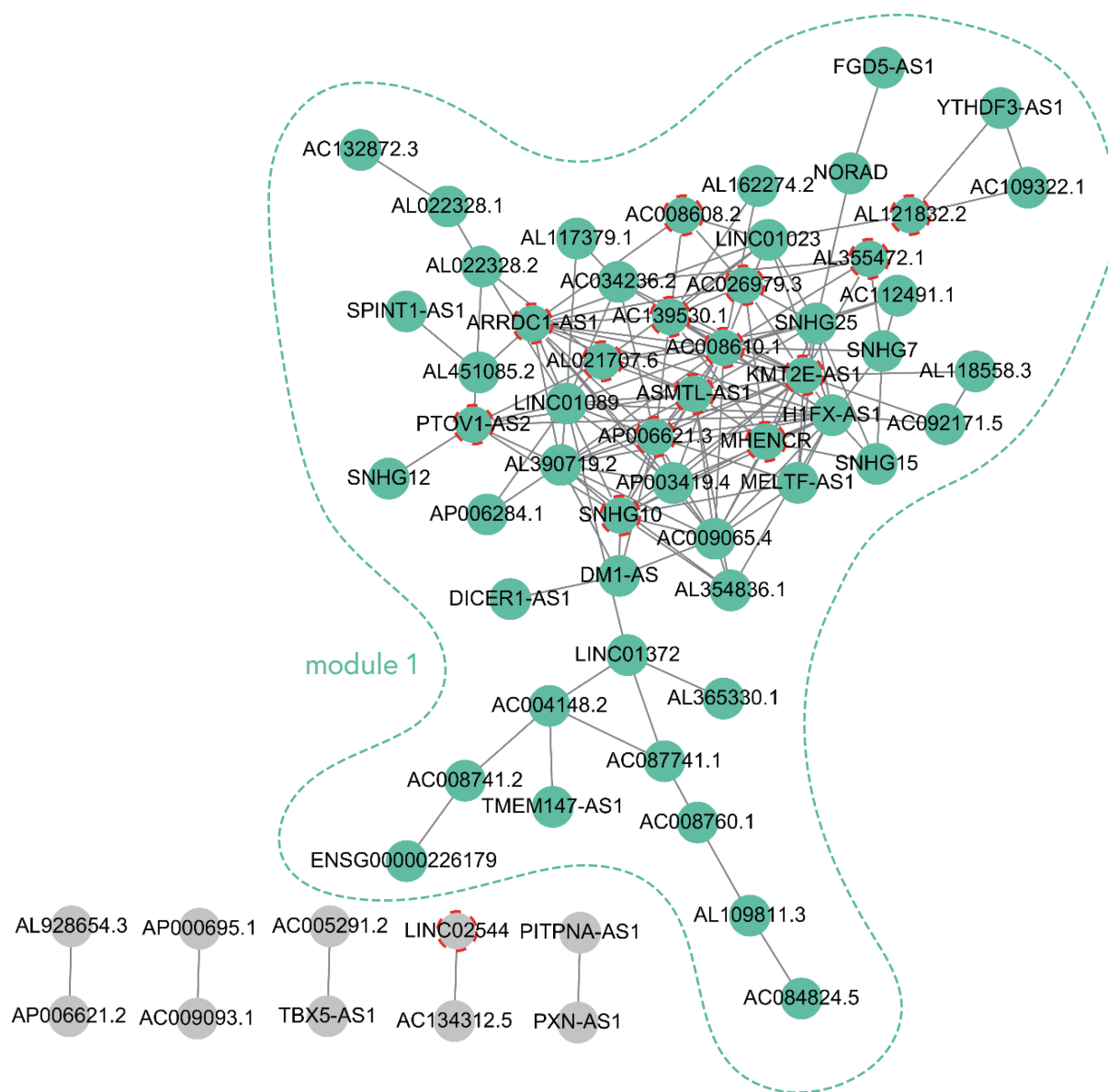

Figure S7.

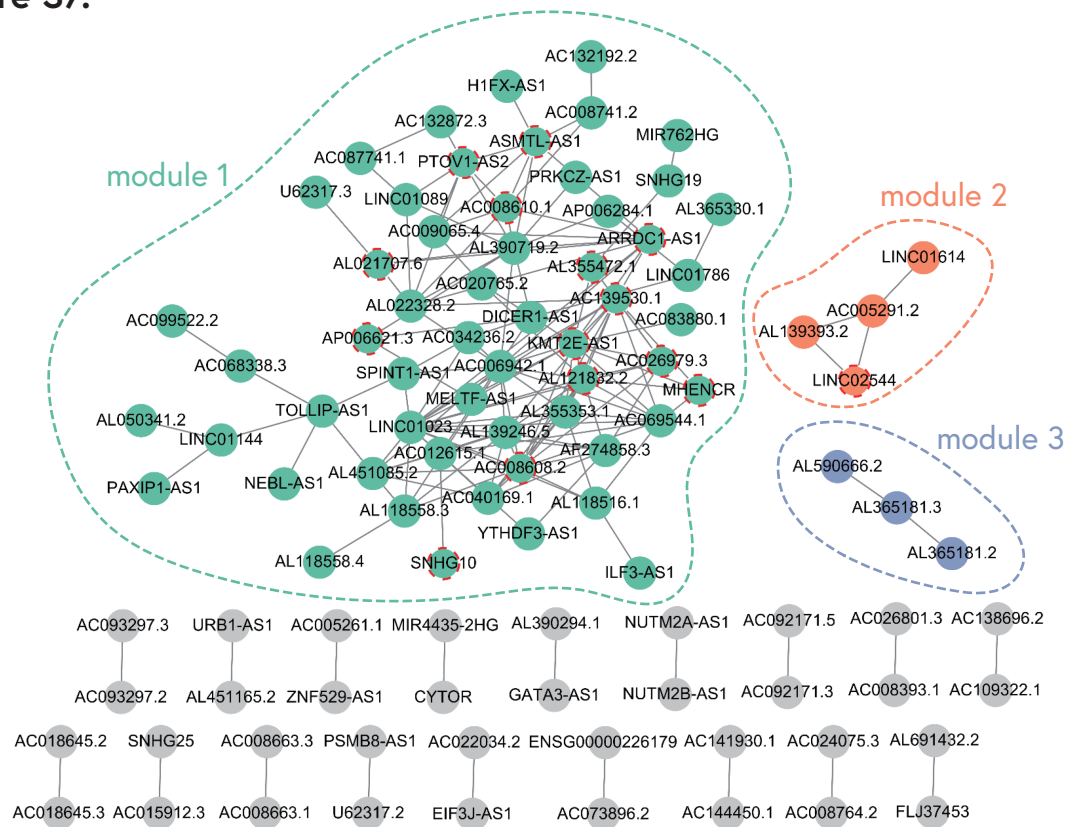

Supplement: Supplementary file 1 — Additional file 1: Figure S1. Distributions of Fisher’s Z transformation for BRCA Her2 type. Figure S2. Distributions of Fisher’s Z transformation for BRCA Luminal A. Figure S3. Distributions of Fisher’s Z transformation for BRCA Luminal B. Figure S4. Illustrations of three kinds of bi-partite co-expression networks and the workflow of the proposed analysis method. Figure S5. Coherent association network in BRCA Her2 type. Figure S6. Coherent association network in BRCA Luminal A. Figure S7. Coherent association network in BRCA Luminal B. [file 12920_2022_1236_MOESM1_ESM.pdf]
